# Revolutionizing medicine: Exploring the breakthroughs in liver xenotransplantation

**DOI:** 10.1016/j.livres.2025.08.001

**Published:** 2025-08-21

**Authors:** Mohamed El-Kassas, Walaa Abdelhamed, Khalid Al-Naamani

**Affiliations:** a Endemic Medicine Department, Faculty of Medicine, Helwan University, Cairo, Egypt; b Applied Science Research Center, Applied Science Private University, Amman, Jordan; c Endemic Medicine Department, Sohag University, Sohag, Egypt; d Department of Medicine, Division of Gastroenterology and Hepatology, The Medical City for Military and Security Services, Muscat, Oman

**Keywords:** Liver xenotransplantation, Genetic engineering, Immune rejection, Coagulation dysfunction, Xenogeneic infections

## Abstract

The critical shortage of liver transplant donors necessitates innovative solutions, with xenotransplantation emerging as a promising alternative. Despite significant ethical, scientific, and practical challenges, recent advancements in liver xenotransplantation, particularly using pigs as donors for non-human primates (NHPs), have extended graft survival duration. However, life-threatening issues such as thrombocytopenia and coagulation disorders persist, limiting survival to under a month. Advances in genetic engineering have enabled the modification of pig genomes to match the human immune system better, targeting genes responsible for immune rejection and increasing compatibility. While these breakthroughs enhance the potential for human transplantation, the challenges of immune rejection and long-term functionality remain substantial. This review highlights recent progress in liver xenotransplantation from pigs to NHPs and explores the implications for potential human clinical application.

## 1 Introduction

Acute liver failure (ALF) and decompensated cirrhosis are treated symptomatically prior to liver transplantation (LT).^1^ Since ALF has a high rate of morbidity and mortality,^2^ intensive care management and emergency LT have improved the overall survival of transplanted patients.^3^^,^^4^ Unfortunately, many liver disease patients die awaiting LT due to rapid disease progression and irreversible neurological damage.^5^ LT could be the only cure option in most cases of ALF, improving the one-year survival rates post-LT to 60%–80%.^6^ Unfortunately, the availability of liver grafts is limited.^7^ Besides ALF, decompensated cirrhosis due to chronic liver diseases, liver-related metabolic disorders, and liver-related malignancies are other common indications for LT in adults.^8^ Globally, the shortage of donor organs remains a significant barrier to successful transplantation.^9^ There are currently over 100,000 people waiting for transplantations in the United States (U.S.) alone, indicating that the demand for organ transplants is far exceeding the supply.^10^ Temporary liver support in ALF patients while awaiting LT is essential to prevent permanent brain damage.^11^ The U.S. Food and Drug Administration (FDA) has approved artificial liver support (ALS) systems, such as the molecular adsorbent recirculating system (MARS), for patients with ALF. Beneficial effects related to specific symptoms were shown with the use of MARS. However, a clear survival benefit has not yet been established.^12^^,^^13^ In addition, ALS technology has not been widely adopted.^14^ The absence of an effective method for temporary liver support has heightened the urgency to develop solutions that can maintain hepatic function during critical periods.^10^ These include hepatocyte transplantation, transplanting hepatocyte-like cells derived from human stem cells that have been expanded, transplanting a transgenic pig liver, *ex vivo* liver perfusion from pigs or non-human primates (NHPs), and scaffold-based transplantation.^10^^,^^11^ This review provides an overview of the history of liver xenotransplantation, highlights recent developments in LT models from pigs to NHPs, and identifies the obstacles that still stand in the way of its clinical application in humans.

## 2 NHPs and wild-type (WT) porcine donors

In anatomical, physiological, and immunologic similarities, NHPs, like baboons, are closer to humans. NHPs such as chimpanzees are also genetically the closest to humans, reducing the chances of graft rejection. However, primates are endangered in the wild, and their use as a source of replacement organs raises ethical concerns because of their high level of intelligence. Therefore, pigs have been selected as the most donor due to their greater physiological compatibility, lower risk of disease transmission, short gestation period, more offspring, growing rapidly, and their organs reach a size matching human organs in three to six months. Additionally, they can be genetically modified with relatively low care expenses. Pigs have become the species most likely to solve the donor organ shortage.^15^ However, pig cells have antigens on their surface, similar to ABO blood group antigens, which trigger a severe immune response called “hyperacute rejection (HAR)”. To overcome this, scientists have inserted human genes into single-cell pig embryos in an attempt to “humanize” their cell-surface proteins, so they are no longer antigenic. Even if this procedure reduces the risk of HAR, other immunological barriers to xenotransplantation will still exist.^15^ The risks of rapid graft rejection and xenogeneic infection transmission are significant concerns for the use of pigs as organ donors.^11^

## 3 History of organ xenotransplantation to human

Blood transfusions from different animal species were used to treat patients with various pathological conditions between the 17th and the 20th centuries.^16^ In the 19th century, skin grafts were also performed using animals; the most common were frogs.^17^ In 1906, Mathieu Jaboulay performed heterotopic transplantation of two renal grafts in humans, using a pig as the donor for one and a goat as the donor for the other. Both grafts failed due to thrombosis.^18^ Reemtsma^19^ transplanted chimpanzee kidneys into 13 patients between 1963 and 1964 when dialysis was not yet common, and human organs were unavailable. In 1964, Hardy^20^ used a chimpanzee heart to perform the first heart transplant on a human, but the patient died in 2 h. Two years later, in 1966, Starzl performed the first liver transplant from a chimpanzee to a human. The discovery of cyclosporine in 1976 revolutionized the field of organ transplantation by providing a powerful immunosuppressive agent, which significantly improved graft survival. However, despite its promise, early attempts at xenotransplantation still faced challenges, as illustrated by the case of Baby Fae—a 12-day-old infant who received an orthotopic baboon heart transplant but succumbed to graft rejection 20 days post-surgery.^21^ Due to this, there was a brief pause in subsequent attempts until the release of tacrolimus in 1992.^22^ In 1992, Starzl^23^ successfully maintained the survival of a patient after LT from a baboon for 70 days. Genetically engineered pigs could provide transplant recipients with an endless supply of organs and cells.^24^

## 4 History of liver xenotransplantation

### 4.1 Trials of liver xenotransplantation from pigs to NHPs

Orthotopic liver xenotransplants from pigs to baboons started in 1968. Among seven transplanted animals, the most extended survival time was 3.5 days. These animals were administered steroids and azathioprine for immunosuppression and eventually died from bronchopneumonia.^25^ Liver biopsies from those animals showed preserved hepatocytes and mononuclear cell infiltration of the portal tracts.^25^ Between 1969 and 1993, a total of 11 hepatic xenografts were performed. The donor animals included seven baboons, three chimpanzees, and one pig.^14^ Two years later, pigs were less successful when transplanted to chimpanzees and rhesus monkeys, and the recipients died in less than 12 h with signs of disseminated intravascular coagulation.^26^ Table 1 summarizes studies on liver xenotransplantation from pigs to NHPs.^25^, ^26^, ^27^, ^28^, ^29^, ^30^, ^31^, ^32^, ^33^, ^34^, ^35^, ^36^, ^37^

**Table 1:** Studies on liver xenotransplantation from pigs to NHPs.

Year	Reference	Donor to recipient	Longest survival
1968	Calne *et al*.^25^	WT pig to baboon	3.5 days
1970	Calne *et al*.^26^	WT pig to rhesus monkey	<12 h
2000	Ramirez *et al*.^27^	CD55 *vs.* WT pig to baboon	8 days *vs.* 8 h
2005	Ramírez *et al*.^28^	CD55/CD59/HT *vs.* WT to baboon	24 h *vs*. <16 h
2010	Ekser *et al*.^29^	GTKO/CD46 *vs.* GTKO to baboon	7 days *vs*. < 1 day
2011	Cowan *et al*.^31^	GTKO/CD46 to baboon	5–7 days
2012	Kim et al.^30^	GTKO to baboon	9 days
2014	Yeh *et al*.^32^	GTKO to baboon	15 days
2015	Ji *et al*.^33^	GTKO to Tibetan macaques	14 days
2016	Navarro-Alvarez *et al*.^34^	GTKO to baboon	7 days
2016	Shah *et al*.^35^	GTKO to baboon	25 days
2017	Shah *et al*.^36^	GTKO to baboon	29 days
2017	Zhang *et al*.^37^	GTKO *vs.* GTKO/CD47 to Tibetan macaques	14 days

Abbreviations: CD55, decay-accelerating factor; GTKO, alpha-1,3-galactosyltransferase gene-knockout; GTKO/CD46, GTKO pigs transgenic for CD46; GTKO/CD47, GTKO pigs transgenic for CD47; HT, H-transferase; NHPs, non-human primates; WT, wild-type.

### 4.2 Gene modification of pigs for liver xenotransplantation

**Fig. 1:**
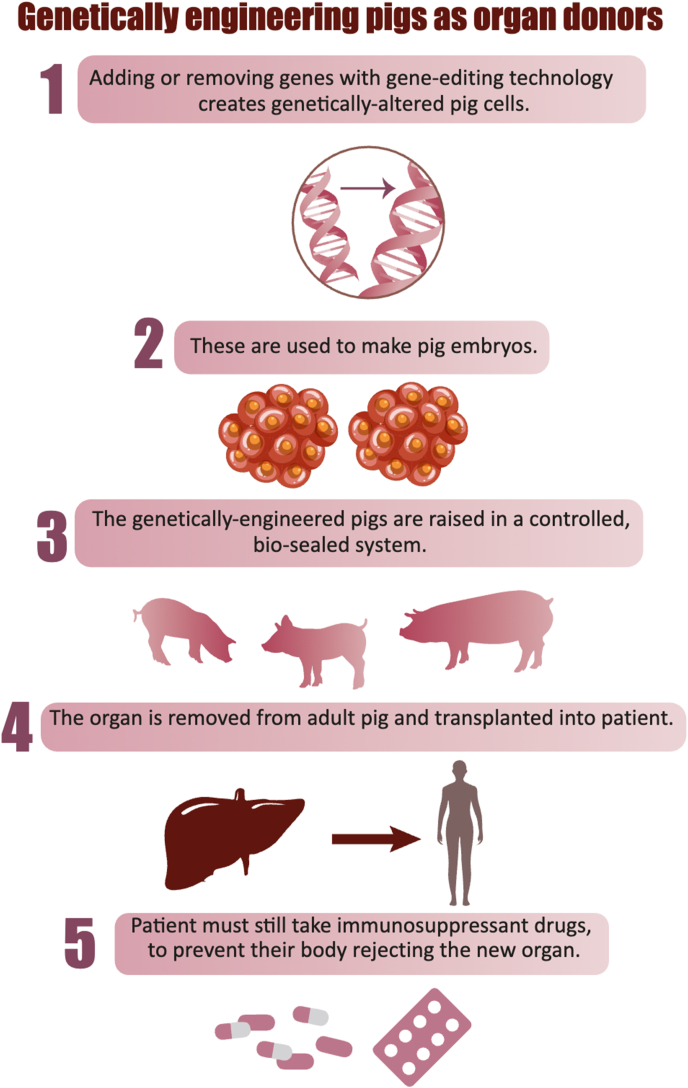
**Genetically engineering pigs as organ donors**. Pigs are genetically modified to serve as potential organ donors for xenotransplantation. Through somatic cell nuclear transfer, pigs undergo genetic alterations aimed at mitigating hyperacute and acute vascular rejection, as well as minimizing the risk of transmitting zoonotic infections (xenozoonosis).

Gene editing has become a rudder for human beings to change the trajectory of life. Since the establishment of the Swine Genome Sequencing Consortium in 2003, exhaustive whole-genome sequences and functional whole-genome annotation systems have been developed,^38^^,^^39^ which have significantly improved the utility of pigs as models. Pigs also have chromosomes that are homologous to those of humans, and the size and composition of their genomes are comparable to those of humans.^40^ It has also been highlighted that the brain, liver, and lymphoid tissues have gene classifications that are highly similar between these two species.^38^ Figure 1 shows genetically engineering pigs as organ donors.

#### 4.2.1 Wild-type (WT) pigs

At the beginning of the 20th century, organ transplantation became possible with the development of vascular anastomosis techniques. In 1993, Makowa and fellows^41^ carried out a heterotopic pig liver xenotransplant to provide temporary metabolic support, intending to execute an orthotopic transplantation as soon as a human donor became available.^41^ Mature WT pigs provided the donor graft. The 26-year-old patient had the highest priority listing with the United Network for Organ Sharing and was admitted to the hospital with fulminant hepatitis, marked encephalopathy, and coagulopathy after a 14-year history of autoimmune hepatitis. In order to reduce the risk of antibody-mediated rejection, naturally occurring anti-pig antibodies were eliminated by plasmapheresis, and *ex vivo* perfusion of the donor pig kidneys was performed preoperatively.^41^ After surgery, the liver xenograft performed well, stabilizing prothrombin levels, producing some bile, reducing lactic acid levels, serum bilirubin levels, and transaminases. Unfortunately, the patient passed away after 34 h due to irreversible brain damage.^41^ A liver biopsy taken 165 min after the transplant revealed signs of endothelial swelling, antibody and complement component deposition, and vascular damage, all of which suggested early graft rejection.^41^ This experience demonstrated that a pig liver can function in a human recipient for a short period and provide some metabolic support in the event of ALF.^11^

#### 4.2.2 Genetically modified pigs

**Fig. 2:**
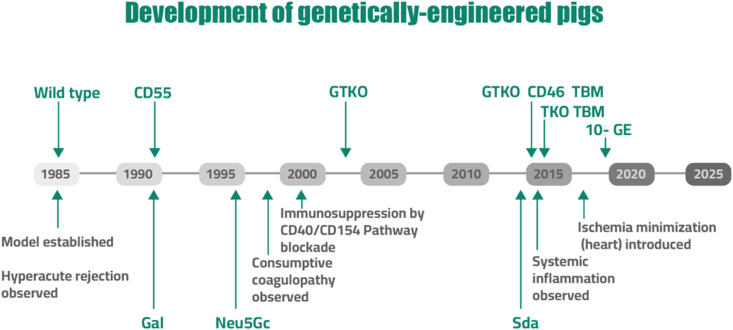
**Serial development of genetically engineered pigs through xenotransplantation**. This figure illustrates the progressive stages involved in the genetic engineering and cloning of pigs, a cumulative effort spanning several decades. Multiple gene deletions and insertions of human transgenes have been systematically applied to surmount immunological and physiological barriers, thus enhancing pig organ compatibility and functionality for transplantation into human recipients. Abbreviations: CD46, membrane cofactor protein; CD55, decay-accelerating factor; Gal, galactose-alpha-1,3-galactose; 10 GE, genetically engineered pigs with 10 genetic modifications, including the inactivation of 4 endogenous porcine genes and insertion of 6 human transgenes; GTKO, alpha-1,3-galactosyl transferase knockout; Neu5Gc, N-hydroxyacetylneuraminic acid; TBM, thrombomodulin; TKO, triple knockout.

The increasing use of gene-editing technologies has renewed interest in liver xenotransplantation.^27^ The first genetically modified pig-to-NHP models focused on expressing humanized complement regulatory proteins (CRPs) in pigs, such as CD55, also known as the decay-accelerating factor, or DAF.^28^ It is theoretically possible to reduce hyperacute antibody-mediated rejection by modifying the complement cascade. When combined with immunosuppressive therapy, two baboon recipients survived for more than four days, longer than any previous attempt using WT pig livers.^27^ Since the first successful preclinical trials for liver xenotransplantation using genetically modified pigs, the number of pigs available for use in xenotransplantation research has increased dramatically.^42^ Xenoantigens, complement pathways, and coagulation cascade components have all been the targets of genetic modifications tested in pig-to-NHP models to extend graft survival.^32^ The first human in China to receive a liver from a genetically modified miniature pig survived for ten days after the transplant, and the recipient was a fifty-year-old man who was clinically dead.^43^ The pig was raised in a dedicated facility free of pathogens, and it tested negative for many infections, including porcine cytomegalovirus, with no symptoms of infection.^43^ Researchers found that clinically dead individuals are not very useful for xenotransplantation because they experience significant hormonal changes once brain activity ceases, as a result, liver xenotransplants are mainly used in patients with acute liver damage as a temporary measure awaiting for a human liver donor.^43^ Another case from China was a 71-year-old man who became the first living human to receive a liver transplant from a genetically modified pig, marking the fifth reported case of a pig organ transplant in a human.^44^ There was a gradual increase in bile production, and the patient survived more than two weeks after the surgery. The hope was that the native liver would regain its function and would be temporarily supported by the xenograft, similar to the concept of auxiliary LT.^44^ Figure 2 shows the serial development of genetically-engineered pigs through xenotransplantation.

## 5 Barriers to xenotransplantation

### 5.1 Infectious agents transmission

**Fig. 3:**
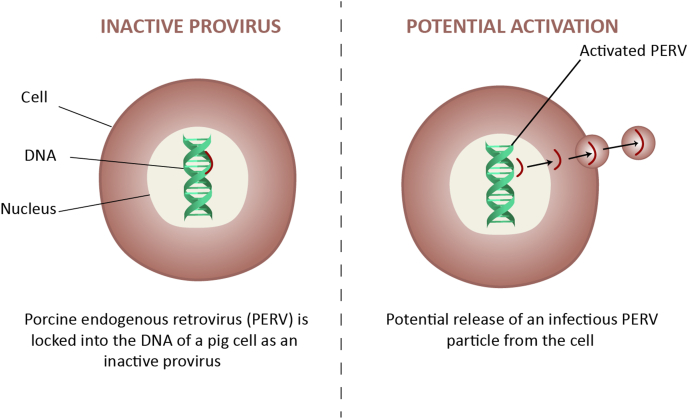
**Porcine endogenous retroviruses (PERVs) activation and spread**. PERVs, which are naturally integrated into the genome of all pigs, represent a critical safety concern in xenotransplantation due to their potential capability to infect human cells. These retroviruses are particularly concerning, given their known association with tumorigenesis and immune system impairments, necessitating strategies for risk management.

A major risk associated with xenotransplantation is the transmission of zoonotic pathogens from a non-human donor, like a pig, to an immunocompromised human recipient.^45^ Porcine endogenous retroviruses (PERVs) have the potential to be transmitted to human recipients, resulting in rapid and persistent replication, leading to tumorigenesis, death, and even spread to the general population.^45^ The use of pigs as organ donors raised serious concerns about the risk of spreading xenogeneic infections, which prompted a moratorium on xenotransplantation and effectively stopped clinical and research activities for more than 15 years at the end of the 20th century until the development of modern gene-editing techniques.^46^ Researchers highlighted the need for more sensitive screening methods for infections.^47^ Consequently, exclusion lists containing pathogenic organisms that could be transmitted from pigs to humans through xenotransplantation have been established.^48^ Strictly selecting “pathogen-free” pigs is the current approach to prevent infectious disease transmission from pig to human in xenotransplantation.^49^ Herd isolation, continual source animal monitoring, exact breeding record keeping, microbiological assessments, and routine veterinary care are used to ensure the general health of donor pigs.^50^ The clustered regularly interspaced short palindromic repeats (CRISPR)/Cas system is the preferred method of gene editing due to its effectiveness, simplicity, adaptability, and relatively low cost compared to other methods like zinc-finger nucleases (ZFNs) and transcription activator-like effector nucleases (TALENs).^51^ Using two CRISPR-Cas9 guided RNAs that targeted the *pol* gene in PERVs, scientists were able to completely eradicate PERVs from an epithelial kidney cell line.^52^ Two years later, they reported cloning the first PERV-free pigs.^53^ However, PERV transmission risk to humans remains controversial.^14^^,^^54^ Figure 3 shows PERVs activation and spread.

### 5.2 Hyperacute cellular rejection

Studies have reported that the rejection of xenotransplanted organs appears histologically different from that of organs transplanted from human donors, and this could enable researchers to predict rejection and create customized immunosuppressant regimens for future xenotransplantation.^47^ Researchers discovered that primary complement system activation via a different pathway was the cause of rejection in guinea pigs used in rat xenotransplantation models.^55^^,^^56^ When organs from WT pigs are transplanted into NHPs or humans, the host’s immune system reaction to the xenograft is due to cell-to-cell interactions secondary to molecular incompatibilities between species. This reaction is known as rapid, antibody-dependent, complement-mediated HAR, similar to the reaction observed when ABO-incompatible allografts are transplanted between humans.^57^^,^^58^

**Fig. 4:**
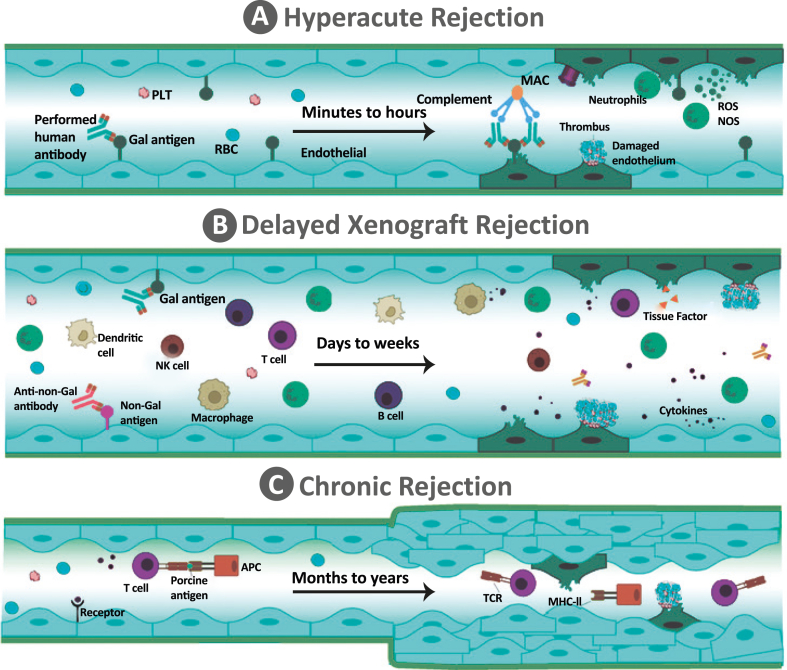
**Types of rejection after xenotransplantation**. This figure summarizes the spectrum of rejection types observed following xenotransplantation. Xenografts may experience vascular rejection driven by anti-donor antibodies and cellular-mediated rejection orchestrated by T lymphocytes. Specifically, hyperacute rejection occurs rapidly within minutes post-transplantation when antigens are entirely incompatible, acute rejection manifests typically from one week to three months post-transplantation, and chronic rejection can gradually develop over several years. Abbreviations: APC, antigen presenting cell; Gal, galactose-alpha-1,3-galactose; MHC, major histocompatibility complex; NK, natural killer; NOS, nitric oxide synthase; PLT, platelet; RBC, red blood cell; ROS, reactive oxygen species; TCR, T cell receptor.

Clinical and histological features indicate that rejection is driven by preformed antibodies and complement acting on specific epitopes present on the pig’s vascular endothelium.^59^ In this case, the main source of rejection is the loss of carbohydrate molecules on porcine cells during the transition from pigs to NHPs and humans.^59^^,^^60^ The majority of human complement-fixing xenoreactive natural antibodies (NAbs) target the lower mammalian cells’ saccharide galactose-alpha-1,3-galactose (Gal).^61^ Despite lacking the Gal epitope, humans and Old-World monkeys have high levels of anti-Gal antibodies due to the presence of gastrointestinal tract bacteria that express the *Gal* gene.^62^ Following xenograft perfusion, NAbs attach to Gal on porcine liver sinusoidal endothelial cells (LSECs), activating the coagulation system and the classical complement pathway leading to graft HAR.^62^^,^^63^ Nevertheless, the donor pig underwent genetic engineering to produce CRPs, coagulation cascade proteins, and multiple other genes while suppressing the expression of important xenoantigens. So far, multiple methods have been implemented to increase graft survival in pig-to-NHP models; these include xenoantigen-targeted gene editing, complement pathway modifications, coagulation cascade components, and immunosuppression.^64^ The removal of Gal, one of the most significant pig antigens contributing to graft failure, significantly advanced genetic remodeling. The creation of alpha-1,3-galactosyl transferase knockout (GalTKO; GTKO) miniature pigs to neutralize the effects of natural anti-Gal antibodies has enabled the testing of pig organ xenotransplantation in pig-to-NHP models.^65^ Further genetic modifications are required due to the persistent evidence of a non-Gal antibody response, even with the use of GTKO pigs, where the Gal epitope has been completely removed from pig organs.^66^ Porcine LSECs are shielded from human complement-mediated damage by transgenic pigs that express human CRPs.^67^ Humanized CRPs, including membrane inhibitor of reactive lysis (CD59), membrane cofactor protein (MCP or CD46), and decay-accelerating factor (DAF or CD55), were used in early genetically engineered pig-to-NHP models to target the complement cascade specifically.^27^^,^^28^^,^^67^ Figure 4 shows types of rejection after xenotransplantation. Later on, LT was performed on two baboons using pigs transgenic for human DAF (hDAF; hCD55); one animal died on day 4 from aspiration-related cardiac arrest, and the other was sacrificed on day 8 due to sepsis and coagulopathy.^27^ Baboons did not experience HAR in the livers of hDAF transgenic pigs, and the porcine liver continued to maintain adequate coagulation and protein levels in the baboon for up to eight days following LT.^27^ Additionally, five baboons were xenografted with multi-transgenic pig livers that expressed human 1,2-fucosyltransferase (H-transferase, HT), hCD55, and hCD59 in order to prevent HAR and enhance liver function. The transplanted livers were able to synthesize coagulation factors, leading to a gradual increase in prothrombin activity to over 35%, with the animals surviving for 13–24 h. In contrast, four baboons transplanted with unmodified pig livers experienced HAR, with survival rates of less than 16 h and no liver function.^28^ The immunosuppressive protocol, which primarily consisted of thymoglobulin induction and tacrolimus, mycophenolate mofetil, and methylprednisolone maintenance, was used to transplant livers from GTKO (*n* = 1) or GTKO pigs transgenic for CD46 (GTKO/CD46, *n* = 5).^68^ The recipients died 4–7 days after transplantation from severe bleeding, thrombocytopenia, and thrombotic microangiopathy (TMA). It was thought that the causes of this dysregulated coagulation were low levels of anti-non-Gal antibody, which activates the porcine vascular endothelium, and underlying species variations in baseline coagulation factor activity.^30^^,^^68^ Besides, three baboons were transplanted with GTKO with an immunosuppressive regimen including thymoglobulin, cobra venom factor, anti-CD154, and azathioprine as induction with maintenance therapy of tacrolimus and a tapering dose of methylprednisolone. However, coagulopathy and sepsis occurred and were the cause of death after 9 days.^30^ Unfortunately, even with the development of hDAF, hCD59 transgenic, and GTKO pigs, the maximum survival for liver xenografts for the previous 50 years up until 2016 had been limited to 9 days.^13^

### 5.3 Thrombocytopenia

The available literature suggests that a combined immune response involving both the innate and adaptive immune systems may exacerbate consumptive coagulopathy and thrombocytopenia, which are commonly observed in liver xenotransplantation cases where there is species discordance, despite the fact that HAR has been successfully treated.^50^ When pigs are transgenic for hCD46 and undergo pig-to-NHP orthotopic liver xenotransplantation, the primary barrier to success has always been severe and fast thrombocytopenia, which appears within an hour, even when there are no signs of humoral or cellular rejection,^29^ along with uncontrollable coagulation dysregulation,^31^ which ultimately leading to fatal hemorrhage in the recipient.^11^^,^^50^ According to Chihara *et al*.,^69^ CD18 is essential for recognizing human platelets by pig Kupffer cells (KCs) and the *in vitro* sequestration of platelets. Anti-CD18 antibodies and CD18 siRNA knockdown in pig cells decreased human platelet binding and phagocytosis.^69^ Asialoglycoprotein receptor-1 (ASGR1)-mediated platelet phagocytosis has been suggested as an additional mechanism of species-discordant platelet consumption.^70^ The macrophage is prevented from phagocytosing by the appropriate species-specific binding of CD47 to signal regulatory protein alpha (SIRPa),^71^ and transgenic expression of human SIRPa on porcine KCs significantly reduced human platelet phagocytosis.^72^ It has been suggested that xenoreactive antibodies within platelet α-granules may bind to recognized xenoantigens of pig liver stem cells (LSCs), allowing platelets to be phagocytosed and causing thrombocytopenia after liver xenotransplantation.^73^ Consequently, it has been proposed that by blocking the coagulation cascade, species incompatibilities in the thrombin-thrombomodulin (TM) complex may also contribute to the dysregulation of coagulation in liver xenotransplantation.^74^ Positive outcomes are noted when human thrombomodulin (hTM) is expressed transgenically.^74^ This expression was lowest in the liver.^75^ While it has shown prolonged survival in preclinical hTM pig-to-NHP kidney transplantation and heart xenotransplantation.^76^^,^^77^ Porcine von Willebrand factor (pvWF) has been shown to bind to human glycoprotein Ib (GpIb) more firmly than human GpIb, which leads to noticeably higher platelet activation *in vitro*.^78^^,^^79^ Moreover, Connolly *et al*.^80^ demonstrated that in the absence of vWF activation, human platelets adhere to and activate spontaneously upon contact with pvWF. One of the reasons for thrombocytopenia and platelet activation seen in xenotransplantation is addressed by the humanized pvWF modification, which expresses humanized pvWF and prevents the improper activation of human or baboon platelets.^80^ Another report showed that porcine LSECs sequester human platelets.^50^ A human prothrombin complex concentrate (hPCC) containing the factors II, VII, IX, and X, and proteins C and S was administered in 2 recipients as a bolus and as a continuous infusion in one baboon, and human recombinant activated factor VII as a continuous infusion in 3 recipients.^35^ Those who received continuous coagulation factor had a significantly lower transfusion requirement, an increase in circulating platelets with increasing doses, and no histological evidence of TMA within the xenografts compared to those who received bolus hPCC, who experienced large and small vessel graft thrombosis with survival of only one to three days. However, the survival was less than seven days.^34^

### 5.4 Coagulation dysregulation

Atypical coagulation cascade activation is one of the primary markers of HAR.^72^ Even in cases where there are no immunologic signs of HAR, dysregulated coagulation occurs in pig liver xenotransplantation to NHP.^72^ The challenge of coagulation dysregulation to liver xenotransplantation is significantly higher than that of kidney or heart xenotransplantation.^81^ The primary reason for consumptive coagulopathy linked to liver xenotransplantation is tissue factor (TF) genetic discordance, which activates the extrinsic pathway.^82^ In the presence of systemic inflammation, TF is expressed in endothelial cells and constitutively expressed in muscle cells and subendothelial fibroblasts.^83^ TF is controlled by the polypeptide known as tissue factor pathway inhibitor (TFPI), which binds to the TF/VIIa complex and inhibits its activity.^83^ Recombinant pig TFPI and human TFPI both successfully prevented human TF/VIIa from activating human factor Xa *in vitro*, according to Lee *et al*.^84^ Furthermore, Ji *et al*.^33^ reported that pig TFPI does not inhibit human TF as effectively as human TFPI does. This implies that molecular variations between pigs and humans may cause coagulation dysregulation following liver xenotransplantation.^33^ Moreover, through activating protein C, blocking factors Va and VIIIa, and reducing the coagulation cascade, species incompatibilities in the thrombin-TM complex have been proposed as a potential contributing factor to the dysregulation of coagulation in liver xenotransplantation.^74^ Based on an *in vitro* study, porcine TM binds to human thrombin; however, the resulting thrombin-TM complex is ineffective in activating human protein C.^74^ The transgenic expression of hTM in porcine aortic endothelial cells resulted in a notable increase in the activation of human-activated protein C *in vitro*, which provides positive insights into the possible uses of hTM transgenic pigs.^85^ Organs from hTM transgenic pigs were found to activate protein C at a significantly higher rate than those from WT pigs; the liver expressed this protein the least.^75^

### 5.5 Liver xenograft dysfunction

One of the obstacles related to liver xenotransplantation is determining whether the transplanted organ will function well enough to support human life.^68^ In 2010, the Pittsburgh Group evaluated a wide range of liver xenograft functional parameters in a series of orthotopic liver xenotransplants from pigs to baboons. These parameters included pig albumin, fibrinogen, haptoglobin, plasminogen, almost all coagulation factors, bilirubin, and all liver enzymes.^68^ Hepatic synthetic function was preserved overall, according to measurements of liver enzymes and international normalized ratio (INR) during recovery.^68^ Kim *et al*.^30^ have documented the need for ongoing intravenous albumin infusion and noted the reduced levels of porcine coagulation factor.

## 6 Costimulation blockade regimens

Interestingly, monoclonal antibodies targeting the CD40/CD40L complex have been shown to prolong liver xenograft survival when combined with costimulation blockade regimens in pig-to-NHP models.^36^^,^^86^ However, no attempts have been made to modify this pathway genetically, probably because pig viability is a concern. When Shah *et al*.^35^ performed an orthotopic pig-to-baboon liver xenotransplantation in 2016, a significant advancement was made, and the recipient lived for 25 days. Two primary differences from previous attempts were identified as the cause of this prolonged survival: the use of belatacept, a monoclonal antibody (mAb) that targets CD80/86, a receptor involved in T-cell costimulation, and the continuous infusion of hPCC to treat coagulopathy.^35^^,^^50^ After a year, the same research team repeated the study with the same costimulation blockade (belatacept, *n* = 3) or anti-CD40 mAb, keeping other parameters unchanged.^36^ After making this adjustment, two recipients experienced an extraordinary survival period of 25 and 29 days, respectively. In one recipient, the final pathology showed C4d positivity, extensive hemorrhagic necrosis, and a focal cytomegalovirus inclusion. The histology of the other recipient showed C4d negative and no signs of rejection, inflammation, or TMA. The livers used in these trials were from GTKO pigs without additional genetic modifications.^36^^,^^50^ The availability of genetically modified pigs in 2017 led to significant breakthroughs in pig-to-NHP models. Rejection-free survival of liver xenotransplantation was extended from 8 days to 29 days with the avoidance of lethal coagulopathy, using a GTKO pig donor with the administration of belatacept, binding to CD80 and CD86 receptors on T cells, and preventing their co-activation with addition the postoperative coagulation factor complex. This demonstrated that the porcine liver can maintain life in NHPs for several weeks and holds promise for clinical application as a bridge to allotransplantation for patients with acute partial liver failure.^36^ Historically, liver xenotransplantation has lagged behind other solid-organ transplants, even though heart and kidney xenografts have shown longer survival times (up to three years).^87^ The most extended survival in liver xenograft transplant so far is only a month or less.^35^^,^^36^

## 7 Other methods of bridging to successful xenotransplantation

Four additional genetic modifications were applied to miniature pigs: human hemagglutinin-tagged-human heme oxygenase-1 (hHO-1), soluble human tumor necrosis factor receptor I IgG_1_-Fc (shTNFRI-Fc), GTKO, and cytidine monophosphate-N-acet-ylneuraminic acid hydroxylase knockout (CMAH KO).^88^ Co-expression of HO-1 and human A20 (hA20) in multiple genetically modified pigs reduced nuclear factor-kappaB (NF-κB) activation, apoptosis and inflammation. In addition to this modification of donor pigs, immunosuppressants like anti-CD154 mAb or anti-CD40 mAb, which block the CD40/CD40L co-stimulatory pathway, are also essential in reducing inflammation and immune responses to extend the survival of xenografts in liver xenotransplantation.^88^ Besides, technological advancements include mammalian cloning, TALENs, and CRISPR. CRISPR/Cas system has become the platform of choice because it is efficient, versatile, easy to use, and relatively inexpensive.^51^ Increasing human albumin in pigs is another possible future goal since pigs naturally produce much less albumin than humans do. By modifying the physiological baseline levels of porcine albumin, long-term low albumin-related issues, such as ascites in the recipient, could be avoided.^89^ However, it could impact the viability of the donor pig, and further research is needed to investigate this approach.^89^

## 8 Novel immunotherapeutics/drugs developed for xenotransplantation

Blockage of pro-inflammatory cytokines such as tumor necrosis factor (TNF)-α neutralizing Ab (adalimumab), interleukin-1 (IL-1) receptor antagonist (anakinra), and IL-6 antagonist (tocilizumab) has been used in pig to NHP xenotransplantation since increased pro-inflammatory cytokines produced by innate immune cells which significantly contribute to the graft rejection.^90^^,^^91^ To control the B cell immune response, B cell depleting anti-CD20 Abs (namely, rituximab) have been used in kidney, heart, and embryonic pancreatic cell transplantations and have shown beneficial effects in an NHP model.^91^ Because anti-CD20 Abs cannot target bone marrow-resident plasma cells that do not express CD20, it has been recommended that agents that inhibit B cell activation factors or survival factors should be used (e.g., belimumab, tabalumab) for further controlling the humoral immune response.^92^ Besides, suppression of effector T cell response is the most critical factor for long-term graft survival; T cell depleting polyclonal antibodies, such as anti-thymocyte globulins (ATGs), have been used since the mid-1970s, after which began the era of biological immunosuppressant development.^93^ Subsequently, more refined monoclonal antibodies against CD3 (muromonab, OKT3) were developed to selectively deplete T cells.^94^ Also, the calcineurin inhibitors (CNIs) inhibit calcineurin from the dephosphorylating nuclear factor of activated T cells (NFAT) and prevent translocation of NFAT into the nucleus, thereby markedly reducing acute graft rejection.^95^ More recently, immunomodulatory drug developments have focused on the identification of targets that control T cell activation and proliferation, such as co-stimulation blockade (e.g., anti-CD154, anti-CD40, and anti-CD28).^96^

## 9 Global regulatory guidelines for xenotransplantation

In December 2016, the FDA released comprehensive industry guidance titled “Issues Concerning the Use of Xenotransplantation Products in Humans.” This document provided a thorough outline of donor animal welfare, pathogen screening for potential donor animals, monitoring of both donors and recipients, and the preclinical efficacy studies necessary before clinical trials could be approved.^97^ Previously, the FDA prohibited clinical trials until it was shown that recipients had a PERV infection. Although this moratorium was removed the following year, PERV monitoring is still necessary.^98^ Due to the significant risk of infection, the FDA prohibited using primate organs in xenotransplantation in 1999. Until pig-to-NHP models showed a “reasonable expectation for success,” many countries had restricted further human xenotransplantation experiments by the late 1990s.^22^^,^^98^ An international board recommended in December 2000 that a life-supporting pig-to-NHP model have a 60% survival rate in good health for at least three months.^22^ More recent guidelines propose that a survival period of over six months is adequate and safe to consider human trials successful.^22^^,^^99^

## 10 Conclusions

Xenotransplantation represents a groundbreaking frontier in addressing the global organ shortage crisis, particularly for liver transplants. Significant strides have been made in overcoming substantial barriers such as acute rejection, thrombocytopenia, TMA, and concerns about zoonotic infections. Advances in genetic engineering have facilitated the development of pig organs with enhanced compatibility for human use. Despite these efforts, challenges remain in achieving long-term graft survival and fully understanding the immune responses triggered by xenotransplants. While preliminary successes provide hope for liver xenotransplantation as a bridge to allotransplantation, further research is essential to refine immunosuppressive protocols and address persistent complications. Continued interdisciplinary collaboration will be crucial in translating these scientific advancements into clinical realities, ultimately improving outcomes for patients with end-stage liver disease or ALF. The future of xenotransplantation holds significant promise, but careful consideration of ethical, regulatory, and clinical factors will be vital to its success.

## Authors’ contributions

**Mohamed El-Kassas:** Writing – original draft, Writing – review & editing, Supervision, Visualization, Conceptualization. **Walaa Abdelhamed:** Writing – original draft, Writing – review & editing. **Khalid Al-Naamani:** Writing – review & editing.

## Declaration of competing interest

The authors declare that there is no conflicts of interest.
